# Whole-Life or Fattening Period Only Broiler Feeding Strategies Achieve Similar Levels of Omega-3 Fatty Acid Enrichment Using the DHA-Rich Protist, *Aurantiochytrium limacinum*

**DOI:** 10.3390/ani9060327

**Published:** 2019-06-06

**Authors:** Jason D. Keegan, Giorgio Fusconi, Mauro Morlacchini, Colm A. Moran

**Affiliations:** 1 Regulatory Affairs Department, Alltech European Bioscience Centre, A86 X006 Meath, Ireland; jkeegan@alltech.com; 2 CERZOO S.r.l, Via Castellarino, 12 San Bonico, 29122 Piacenza, Italy; giorgio.fusconi@unicatt.it (G.F.); mauro.morlacchini@unicatt.it (M.M.); 3 Regulatory Affairs Department, Alltech SARL, Vire, Rue Charles Amand, 14500 Vire, France

**Keywords:** broilers, DHA, omega-3, fatty acids, enrichment

## Abstract

**Simple Summary:**

In many parts of the world, the human population does not consume sufficient quantities of omega-3 fatty acids. Humans can potentially reduce the risk or severity of a variety of illnesses by simply increasing their dietary intake of omega-3 fatty acids, with eicosapentaenoic acid (EPA) and docosahexaenoic acid (DHA) being particularly beneficial. Fish are a rich source of these important fatty acids, but many individuals do not consume fish and so the enrichment of more commonly consumed foods has been explored as a method to increase the consumption of omega-3 fatty acids. The fatty acid content of chicken meat reflects the fatty acid composition of their diet and so poultry meat can be easily enriched by introducing omega-3 rich ingredients into poultry diets. In this study we fed broilers diets supplemented with a DHA-rich protist, *Aurantiochytrium limacinum* for their whole life (42 days) or for the final 21-day fattening period to investigate which strategy represented a more efficient use of the DHA-rich ingredient. As similar levels of enrichment were found from both feeding durations tested, the study indicates that feeding for the 21-day fattening period is the more efficientperiod of dietary inclusion for *Aurantiochytrium limacinum*.

**Abstract:**

The fatty acid composition of broiler chicken tissues can be increased by adding omega-3 rich ingredients to their diets. The purpose of this study was to compare the levels of tissue enrichment observed following the supplementation of broilers with the docosahexaenoic acid (DHA)-rich protist, *Aurantiochytrium limacinum* (AURA) for their whole life (42 days) or for the final 21-day fattening period. Day-old chicks (n = 350) were distributed among 35 pens (10 birds per pen) with each pen randomly assigned to one of five treatments: Control; 0.5% AURA from day 0–42; 1% AURA from day 0–42; 0.5% AURA from day 21–42; 1% AURA from day 21–42. Production parameters were recorded over the course of the study and the fatty acid profile of the breast, thigh, liver, kidney and skin with adhering fat was quantified at the end of the feeding period. The level of supplementation had a significant impact on the degree of omega-3 tissue enrichment, however, no differences were observed when the same dose was provided for 21 or 42 days. These results indicate that supplementation with AURA for a period of 21 days does not negatively affect broiler productivity and is the most efficient strategy to increase the nutritional value of broiler products.

## 1. Introduction

Increasing consumer awareness of the health benefits associated with the consumption of omega-3 polyunsaturated fatty acids (n-3 PUFA) has resulted in a greater demand for n-3 PUFA rich produce [1]. As a result, the industry has been developing strategies to increase the n-3 PUFA content of commonly consumed foods [2]. By feeding chickens diets rich in n-3 PUFA, the fatty acid content of chicken meat can be altered to contain much higher quantities of n-3 PUFA, which can lead to health benefits for the consumer without making significant changes to their diet [2]. There are a variety of n-3 PUFA rich ingredients that have been used to supplement poultry diets in order to increase the n-3 PUFA content of their meet and eggs. Linseed, in various forms, can be a good source of alpha-linoleic acid (ALA) and can significantly increase tissue n-3 FA content [3]. However, some antinutritional features of linseed limit its inclusion in poultry diets [3]. In addition, most of the health benefits associated with omega-3 fatty acids have been attributed to increasing the intake of eicosapentaenoic acid (EPA) and docosahexaenoic acid (DHA) [4]. While the human body can convert ALA to EPA and DHA, this process is inefficient and as such, the direct consumption of at least 250 mg of EPA+DHA per day is recommended [5]. Fish are a rich source of EPA + DHA but are only consumed regularly by small proportions of the population [6]. For those who do not consume fish, chicken has been found to contribute up to 28% of the dietary intake of EPA+DHA in the UK [7] and 24% of EPA + Docosapentaenoic acid (DPA)+DHA intake in Australia [8], despite the fact that chicken is not a naturally rich source of n-3 PUFA [9].

Fish meal and oil can also be used as a feed ingredient for poultry [10], but while this does increase the EPA + DHA content of the meat, it can also lead to a deterioration in consumer acceptability [11,12]. In addition, using fish meal/oil to supplement animal diets is not considered sustainable and could not supply the n-3 PUFA requirements of the world’s growing population [13].

*Schizochytrium* spp. and *Aurantiochytrium limacinum* are both members of the Thraustochytrid family of heterotrophic protists, commonly classified as microalgae, but with fungus-like characteristics. The Thraustochytrids are primary producers of n-3 PUFA in the marine food chain and can be grown in a sustainable manner on a large scale using heterotrophic fermentation. Various authors have used *Schizochytrium* as a dietary supplement for broilers, and have detected increased tissue n-3 PUFA concentrations without any major impact on the organoleptic properties of the meat or on broiler productivity [14,15]. *A. limacinum* (AURA) is a closely related species that can be provided as a dietary supplement and has been shown to increase the DHA concentrations of cows milk, pork, chicken meat and eggs [16,17,18,19,20,21].

Previous work indicated that AURA is well tolerated by broilers with no negative impact on broiler health or productivity observed when broiler diets were supplemented at a level of 5% for their whole life [16]. Significant increases of tissue DHA concentrations were also observed when feeding broilers for the final 21-day fattening period only [17]. When comparing these similar studies, the results suggested that feeding for the entire life of the broiler may represent a more cost efficient use of dietary AURA, reaching higher levels of enrichment potentially due to the accumulation of DHA in the tissues over the whole life of the bird [17]. These comparisons were drawn from studies of similar design, carried out in the same facility, but at different times. As the studies did not occur concurrently, differences between the levels of enrichment could be attributed to factors other than the duration of supplementation. Therefore, in the current study we aimed to investigate the most cost-effective duration of supplementation by comparing the levels of enrichment achieved when birds were provided with supplementary AURA for their whole lives or for the final 21-day fattening period only.

## 2. Materials and Methods

The research protocol and animal care were conducted in accordance with European Union Directive 2010/63/EU covering the protection of animals used for experimental or other scientific purposes, and according to the recommendation of the Commission of 2007/526/CE covering the accommodation and care of animals used for experimental and other scientific purposes. The live animal portion of the study was carried out at the CERZOO research center which is authorized by the Italian Ministry of Health to employ animals for experimental or other scientific purposes. The study was conducted to investigate the effect of supplementing broiler diets with *A. limacinum* (AURA; ALL-G-RICH^®^; CCAP 4087/2; Alltech Inc., Nicholasville, KY, USA) over their whole life (WL; Day 0–42) or for the 21-day fattening period only (FP; Day 21–42) on production parameters and the fatty acid content of breast, thigh, liver, kidney and skin samples. The analytical composition of AURA was determined prior to the start of the study using the following methods: crude protein (AOAC 990.03), crude fat (AOAC 954.02), fatty acid composition (AOAC 996.06, with matrix validation [22]), moisture (AOAC 930.15) and ash (AOAC 942.05).

The study was carried out using male Ross 308 broilers housed in 1.4 × 1.6 m pens. Food and water were provided ad libitum using one feeder per pen and using an internal water system network. The study site was equipped with a dynamic ventilation system. Heating was provided from positive water aerotherms and positive pressure ventilation was achieved by single, variable-speed fans linked to temperature sensors. The temperature program was recommended by the breeder [23] and was automatically controlled and programmed to gradually reduce the temperature as follows: 25–30 °C (D1); 22–27 °C (D1–D7); 19–25 °C (D7–D14); and 18–25 °C (D14–D42). The higher temperature at the beginning of the study was guaranteed by specific 150-watt lamps mounted in each pen. The relative humidity ranged from 60–80% (D1–D7) and 40–80% (D7–D42). The lighting program was 18 hours light, and 6 hours dark.

Day-old chicks (n = 350) were randomly distributed among 35 pens (10 birds per pen) with each pen randomly assigned to one of the five following treatments: 0% AURA day 0–42 (Control); 0.5% AURA from day 0–42 (0.5%WL); 1% AURA from day 0–42 (1%WL); 0.5% AURA from day 21–42 (0.5%FP); 1% AURA from day 21–42 (1%FP). The ingredients of the diets are shown in Table 1 and were designed to meet the nutrient levels recommended for Ross 308 broilers [24]. The experimental diets were produced at the CERZOO feed mill and provided as a crumble from day 0–10, or as pellets from day 10–42. The nutrient content of the experimental diets was determined at the Institute of Food Science and Nutrition (Faculty of Agricultural Sciences, Food and Environment, Catholic University of Sacred Heart, Piacenza, Italy) using the following methods: dry matter (ISO 6496); crude protein (Gazzetta Ufficiale Serie Generale n.92 of 21.04.96); crude fat (Directive EEC 84/4/EEC); crude fibre (Directive EEC n.92/89); crude ash (NEN 3329, ISO 5984-2002 (E)); starch by polarimetric method (Directive CEE n. 72/199 and ISO 10520:1997 (E)); sugars ( Gazzetta Ufficiale CEE n. L155 of 12.07.71); energy content was calculated according to the equation proposed in G.U. CE n. L54 22.02.09. Individual fatty acids were quantified using AOAC Met. 996.06.2001, recently validated for chicken feed [25]. In brief, the fatty acids in each sample were trans-esterified in situ with 1.5 N HCl in methanol, in the presence of toluene. The toluene contained methyl tricosanoate which acted as the internal standard. The resultant fatty acid methyl esters (FAMEs) and toluene were then extracted. The FAMEs were then separated, identified, and quantified by gas chromatography equipped with a flame ionization detector (GC-FID).

The mg of FAME per 100 g of sample was then calculated using the following formula:

$$\mathrm{FAME},\;\mathrm{mg}\;\mathrm{FA}/100\;g\;\mathrm{wet}\;\mathrm{sample}=\frac{A_{X}\times \;CF_{X\;}\times \;W_{IS\;}\;\times 100\;}{A_{IS}\;\times \;W_{S}\;\times \;1.04\;\times \;1000}$$

where A_X_ = area counts for the FA; A_IS_ = area counts for internal standard (C23:0); CF_X_ = instrumental response factor for FA (EPA = 0.98, DHA = 0.99, GLA = 0.98); W_IS_ = weight of IS added to sample in mg; WS = weight of sample in mg; 1.04 = conversion factor from methyl ester to fatty acid.

The AURA used in the study contained 71.7 g of crude fat/100 g biomass and 16.0 g DHA/100 g biomass with a significant amount of palmitic acid (34.0 g/100 g biomass). Additionally, the AURA contained 13% crude protein, 2.63% ash and 1.97% moisture. The analytical composition of the experimental diets is shown in Table 2, while the fatty acid composition of the diets is shown in Table 3. Daily bird health, mortality and culling records were maintained. Pen live weight on days 0, 21 and 42 were recorded in addition to the feed intake and feed refusals. One bird per replicate was killed (according to annex I of the Reg. CE n. 1099/2009 of the council of the 24/09/2009 concerning the protection of animals during slaughter) on day 43 and both kidneys, the whole liver, breast, thigh and skin with adhering fat samples were taken post-mortem. The left breast and thigh were used for the determination of dry matter (UNI ISO 1442), crude fat (AOAC 991.36), protein (UNI ISO 937) and ash (AOAC 991.36). The fatty acid content of both kidneys, the liver and the right (skinless) breast, (skinless) thigh and skin with adhering fat were quantified using the method recently validated for chicken tissues [26]. FAME calculations were as described above for chicken feed.

Differences between the treatment groups were determined using the general linear model procedure of Minitab (Minitab, v18, State College, PA, USA) with Tukey’s post hoc analysis used to determine the differences between the treatment groups. Regression analysis was used to determine whether the estimated DHA intake per bird could predict the DHA content of breast and thigh meat. DHA intake per bird was calculated by dividing the intake per pen by the number of birds present and then multiplying by the DHA content detected for each experimental diet.

## 3. Results

### 3.1. Bird Health and Performance

Mortality was below 5% for each treatment group with an overall mortality of 3.14% observed. The performance of the birds in terms of their weight, weight gain, feed intake and feed conversion ratio (FCR) are summarised in Table 4. There was no significant difference between the groups in terms of weight at the beginning of the study. The 1%WL and 0.5%WL groups differed significantly in terms of weight by day 21 and in terms of their average daily gain during the first period (D 0–21) with the latter gaining significantly more weight. By day 42, the 1%WL group differed significantly to the control in terms of weight and average daily gain, with lower values again observed for the 1%WL group. Average daily feed intake differed between the 1%FP group and the 0.5%WL groups between day 0 and 21 and between the control and 1%WL groups between day 21 and 42, and overall. Thigh weight differed significantly between the control and 1%WL group with the latter weighing significantly less than the control at the end of the trial.

### 3.2. Meat Fatty Acid Content

**Figure 1:**
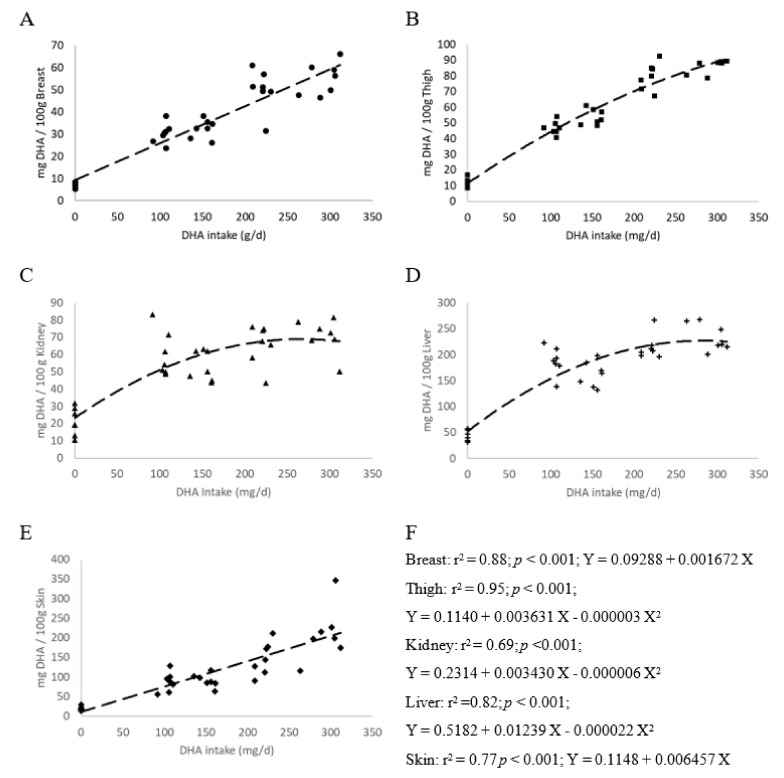
Scatterplots with regression lines for docosahexaenoic acid (DHA) intake (mg/day) against DHA content (mg/100g) detected in breast (**A**) thigh (**B**), kidney (**C**), liver (**D**) and skin with adhering fat (**E**). The r^2^ value, significance of the relationship (*p*) and model equation are shown for each graph in panel **F**.

**Figure 2:**
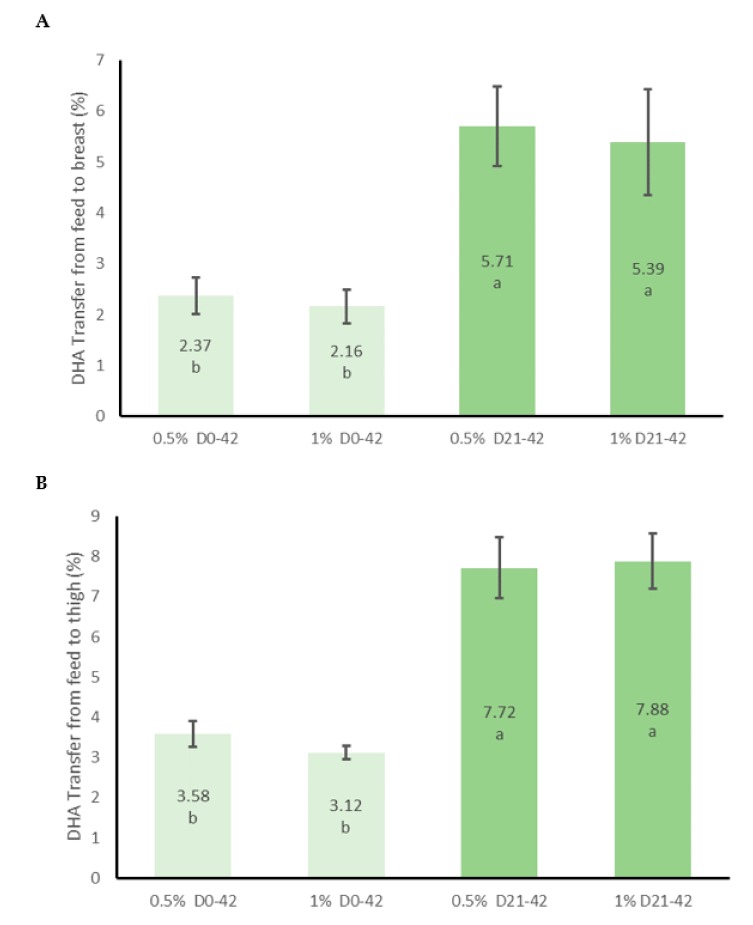
Mean efficiency of DHA transfer (± 95% C.I.) from feed to the breast (**A**) or thigh (**B**) tissues. Transfer efficiency was estimated for one bird per pen (n = 7 per treatment) as follows; (mg DHA in feed × average daily feed intake x 42 days) ÷ (mg DHA/g breast or thigh × breast or thigh weight) × 100. ^a,b^ Columns that do not share a letter differ significantly (*p* < 0.001).

The effects of supplementation with AURA on the concentration of selected fatty acids (i.e., those fatty acids that differed significantly between treatments or were of interest overall) in chicken breast, thigh, kidney, liver or skin with adhering fat are shown in Table 5. The full fatty acid profile determined for each tissue/organ is provided in Supplementary Table S1. The highest DHA concentrations were detected in the liver followed by the skin with adhering fat, thigh and kidney, with the breast meat having the lowest DHA content of all the sampled tissues. In all cases, the DHA content of the tissues/organs of supplemented birds were significantly higher than the unsupplemented control group. For breast and thigh samples both 1% groups had significantly more DHA than all other treatments with similar levels of enrichment observed for the 1%WL and 1%FP groups. The groups supplemented with 0.5% AURA were enriched to a similar degree with no difference observed between the 0.5%WL and 0.5%FP treatments. In the liver, both 1% treatments were again the most enriched with the duration of supplementation not effecting the level of enrichment. In the kidney, the 1%WL and both FP treatments were enriched to a similar degree, with the 0.5%WL group having significantly less DHA than the 1%WL group. In the skin the 1%WL treatment had significantly more DHA than each of the other treatments, with all remaining supplemented groups enriched to a similar degree. A quadratic relationship between DHA intake and tissue/organ DHA content was observed for the thigh, kidney and liver samples, while a linear relationship was observed for breast and skin samples (Figure 1). The efficiency of DHA transfer from the feed to the breast and thigh was estimated and indicated that feeding for the final 21 days resulted in a more efficient transfer of DHA from the feed to the tissues for both breast and thigh (Figure 2).

The concentration of DPA did not differ significantly between any of the treatment groups for any of the tissues/organs sampled. The highest levels of EPA enrichment were observed in the skin with adhering fat, followed by the kidney, liver, thigh and breast. For each tissue/organ sampled the highest EPA concentration was detected in the 1%WL group, with this group having more EPA than the control group in each case. The 1%FP group also had significantly more EPA than the control group in each case with the exception of the breast tissue samples. The 1%FP group was enriched to a similar degree as the 1%WL group in each tissue with the exception of the skin, in which the 1%WL group had significantly more EPA (Table 5). These differences contributed to significant differences between the groups in terms of their total n-3 PUFA concentration, with groups receiving the 1% AURA treatments generally enriched to a greater degree. For each tissue the C22:4n-6 concentration detected in the supplemented groups was generally significantly lower than the control, however, the same trend was not observed for the total n-6 concentrations, which did not differ between the treatment and control groups. The n-6/n-3 ratio for both 1% treatments were significantly lower than the control group in every tissue/organ sample. Both 0.5% AURA treatments also had significantly lower n-6/n-3 ratios in every tissue/organ samples with the exception of the skin.

## 4. Discussion

In our previous studies, the addition of AURA was found to have no effect on any of the broiler productivity parameters measured [16,17]. In contrast, the 1%WL group in the current study was found to have a lower average weight than the control group by day 42, likely due to a significantly lower average daily feed intake between day 21 and 42. For the whole 42 day period, feed intake did not differ between the groups with no differences in feed conversion ratio, European Production Efficiency Factor, dressing % or breast weight observed. In our previous study, investigating supplementation at up to 5% of the diet, no differences in productivity were observed between the groups [16] which may suggest that the differences observed in the current study were not as a result of the inclusion of AURA. Some other n-3 PUFA rich ingredients have been shown to negatively impact broiler productivity, with the anti-nutritional properties of linseed limiting its inclusion in broiler diets [3]. In contrast, some authors have shown improvements in weight gain, feed intake and FCR when providing omega-3 rich *Schizochytrium* [10,27] or AURA [28]. The effects on the 1%WL group could be considered minor and considering there was no differences between the two groups supplemented for the fattening period only, supplementation with AURA is unlikely to reduce the productivity of broilers in practice.

It has previously been demonstrated that supplementing the diets of broilers with AURA is an effective method that can increase the concentration of DHA in broiler tissues [16,17]. When comparing the results of our previous studies, feeding 0.5% AURA for 42 days resulted in a similar level of tissue enrichment as feeding 1% AURA for 21 days [16,17]. Interestingly, the cost of supplementation (with an approximate AURA cost of €7 per kg) for 42 days at a level of 0.5% of the diet (€0.18 per bird) was lower than the cost of providing AURA at a level of 1% of the diet for 21 days (€0.26 per bird). These studies were carried out in the same research facility, over a two-year period using similar diets, however, differences in the level of enrichment observed between the studies could have been caused by factors other than the duration of feeding. As such, it was important to test the effect of AURA feeding duration on tissue DHA enrichment in a single experiment. Here we found that feeding a lower amount of AURA over a longer period did not lead to similar levels of enrichment as feeding higher levels over a shorter period. Furthermore, we found no effect of duration of feeding (i.e., 21 or 42 days) on the level of enrichment observed at each dose. The cost of supplementing the birds for 21 days at 1% of the diet in the current study would be an estimated €0.23 per bird. Feeding at a rate of 0.5% for the whole life of the bird would be less expensive (€0.16 per bird), but the degree of enrichment achieved remained significantly below that of birds supplemented at a rate of 1% for a shorter duration. Feeding for the final 21-day fattening period only is further supported by the significantly higher transfer efficiencies observed for the FP treatments when compared to the WL treatments. These results indicate that the most efficient feeding strategy is to feed for the final 21-day fattening period only.

The level of enrichment observed in the breast samples of birds supplemented with AURA at a rate of 0.5% fell within the range of values reported from our previous studies (23–35 mg DHA/100 g breast) when broilers were supplemented at the same rate. The DHA content of the thigh samples from the current study (47 and 54 mg DHA/100g thigh following 21 and 42 days, respectively) exceeded the range of 24–43 mg DHA/100g thigh reported from our previous studies. A similar trend was observed for the 1% AURA treatments, with the DHA concentration of breast samples from the current study (50 and 55 mg DHA/100 g breast following 21 or 42 days, respectively) and those from the thigh samples (80 and 86 mg/100 g thigh after 21 or 42 days respectively) exceeding the range reported from the breast (36–48 mg DHA/100 g breast) and thigh (42–50 mg DHA/100 g thigh) from our previous trials [16,17]. The fat content of the tissues in the current study was generally higher than those of the tissues from previous studies, increasing their capacity for DHA enrichment. However, the degree of fat deposition has been shown to be unaffected by increasing levels of n-3 PUFA with other nutritional and genetic factors likely responsible for the differences observed [29], between the overall levels of fat detected in the different studies.

Zuidhof et al. [30] investigated the effect of the duration of dietary supplementation of broilers using ground full-fat flaxseed as a source of n-3 PUFA and found that feeding for 24.1 days was the most cost effective duration that achieved an adequate level of tissue enrichment. Kanakari et al. [31] also used flaxseed to supplement broiler diets and showed that feeding for 2–4 weeks prior to slaughter would be sufficient for broilers to accumulate the same amount of n-3 LCPUFA as being fed for six weeks, which is similar to the findings of the current study. When supplementing the diets of broilers with fish oil accompanied by either flaxseed, lard or rapeseed, Konieczka et al., [32] found that feeding for a duration of three weeks before slaughter resulted in significantly higher EPA + DHA concentrations than feeding for one or two weeks only. Our previous work in laying hens indicated that supplementation at a level of 1% of the diet increased the DHA content of eggs for the first 24 days, with no significant increase in the concentration of egg DHA after that point [18]. Overall, these results, using a variety of different n-3 PUFA sources, indicate that feeding for periods longer than 2–4 weeks is not a cost-effective use of the supplemental ingredient.

For a food to be labelled “a source of omega-3” in the European Union (EU) it must contain at least 40 mg EPA+DHA/100 g, while to be labelled “high in omega-3” it must contain 80 mg EPA + DHA/100 g [33]. For the breast meat, both the 0.5%WL and the 0.5%FP groups fell just short of this target with 35 and 33 mg EPA + DHA/100g detected respectively. The thigh samples however, did meet these criteria with 59 mg and 51 mg EPA + DHA/100 g detected in the 0.5%WL and 0.5%FP groups respectively. The breast samples of the 1%WL and 1%FP groups met the criteria to be considered a source of omega-3, with 60 and 54 mg EPA+DHA/100 g detected respectively, while the thigh samples could be considered high in omega-3 with 94 and 87 mg EPA+DHA/100 g detected respectively. Based on these results, the broilers would need to be supplemented at a rate of 1% of the diet so that both the breast and thigh meat could be labelled as “a source of omega-3” in the EU.

No EPA was detected in any of the diets provided, but despite this, an increased inclusion of AURA generally led to increased tissue concentrations of EPA. Similar results have been obtained in our previous trials [16,17], with the increase likely due to the retro-conversion of DHA to EPA [34]. In contrast, no differences were observed between the treatments in terms of the tissue n-3 DPA concentration. This is in keeping with the results of our previous study, when supplementing broilers with 0, 0.25, 0.5 and 1 % AURA, resulted in no differences in n-3 DPA concentrations between treatments in breast tissue samples, while in the thigh samples, the 0.5% treatment had significantly less n-3 DPA than the control group with no differences observed between the other groups [17]. Small but significant increases in breast n-3 DPA were observed when birds were supplemented with AURA at a rate of 2.5% and 5% of the diet with 10.19 and 10.52 mg n-3 DPA/100 g detected respectively, while at 0 and 0.5% of the diet 9.02 and 9.02 mg n-3 DPA/100 g were detected respectively. It does not appear that this increase in tissue n-3 DPA is due to some form of retro-conversion from DHA. It could more likely be a result of the elongation of EPA, which was present in higher concentrations in the 2.5 and 5% treatments. The elongation of EPA to n-3 DPA is more efficient than the elongation of EPA to DHA [35]. In humans, supplementary DHA from an algal source (containing no EPA) was found to significantly increase blood EPA concentrations while reducing the concentration of n-3 DPA indicating that DHA supplementation could negatively influence the concentration of n-3 DPA, possibly as result of competition at the level of fatty acid esterification [36]. These findings indicate that supplementary DHA-rich AURA, when provided at a level of 0.5–1% of the diet can also increase the tissue concentrations of the beneficial fatty acid EPA, despite it being absent from the experimental diets.

The significant increases in both EPA and DHA contributed to the higher total n-3 PUFA concentrations detected in many of the tissue/organ samples. The concentration of C20:4n6 was significantly lower in most of the tissue/organ samples from supplemented birds, however, this did not translate into lower total n-6 PUFA concentrations in any of the tissues/organs. Importantly, supplementation led to lower n-6/n-3 ratios, in every treatment and in every tissue/organ with the exception of the WL skin with adhering fat treatments. Reductions in a person’s overall n-6/n-3 ratio have been shown to improve the health outcomes of patients suffering from a variety of illnesses, including cardio-vascular diseases and some forms of cancer, with the effective ratio based on the type of illness in question [37]. Consuming this AURA enriched broiler meat could help to reduce the overall n-6/n-3 ratio of a person’s diet.

No attempt was made to assess the effect of supplementation on the consumer acceptability of the meat of supplemented birds. However, supplementing broiler diets with marine protists at a level of 2.8% of the diet was previously found to have no impact on consumer acceptability [14]. As the inclusion rates observed in this trial were below this, it is unlikely that the consumer acceptability would be affected. However, it would be beneficial to determine the consumer acceptability of these enriched meats when fresh and following a period of storage. Recent work investigating the frozen storage stability of DHA found significant reductions in the tissue DHA content after a period of 12 or 24 weeks [26]. Chicken meat for human consumption is unlikely to be stored for this extended period of time and over this duration the DHA concentration of breast meat decreased by 35%, 35%, 29.4% and 27.8% in broilers supplemented with AURA at a rate of 0%, 0.5%, 1.5% and 2.5% of the diet. As such, it would be beneficial to establish the effects of storage on the stability of DHA and the consumer acceptability of the AURA enriched broiler meat over a shorter time period.

## 5. Conclusions

These results indicate that providing AURA as a dietary supplement for broilers for the final 21-day fattening period can improve the nutritional quality of broiler meat by significantly increasing the DHA and EPA concentrations of tissues and organs, as well as lowering the n-6/n-3 ratio without significantly impacting productivity.

## Figures and Tables

**Table 1:** Ingredient composition of the five experimental diets supplemented with 0, 0.5 or 1% *Aurantiochytrium limacinum* (AURA) for their whole life (WL; day 0–42) and for the fattening period only (FP; day 21–42).

Diet Ingredient	Day 0–21			Day 21–42		
	0%WL 0.5%FP 1.0%FP	0.5%WL	1.0%WL	0%WL	0.5%FP 0.5%WL	1.0%FP 1.0%WL
Corn meal	46.20	46.50	46.25	52.96	52.90	52.90
Soybean meal 48%	36.70	36.58	36.60	30.12	30.00	29.88
Wheat meal	8.25	8.00	8.00	8.55	8.53	8.50
Soybean oil	4.50	4.13	3.90	4.58	4.28	3.93
Dicalcium phosphate	1.80	1.76	1.75	1.37	1.37	1.37
Limestone	1.00	0.98	0.97	0.98	0.98	0.98
Sodium bicarbonate	0.40	0.40	0.39	0.39	0.39	0.39
Lysine HCL	-	-	-	0.10	0.10	0.10
DL - Methionine	0.40	0.40	0.39	0.20	0.20	0.20
Vitamins and minerals ^1^	0.50	0.50	0.50	0.50	0.50	0.50
Salt	0.15	0.15	0.15	0.15	0.15	0.15
Ronomix Hiphos	0.10	0.10	0.10	0.10	0.10	0.10
AURA	-	0.50	1.00	-	0.50	1.00

^1^ The content of vitamins and trace minerals per kg of premix (Rovimix B extra /1), produced by DSM Nutritional Product—Istituto delle Vitamine, Segrate (MI), Italy, is as follows: vit. A: 2,000,000 IU/kg; vit. D3: 600,000 IU/kg; vit. E: 15,000 mg/kg; vit. K3: 612 mg/kg; vit. B1: 400 mg/kg; vit. B2: 1200 mg/kg; D-pantothenic acid: 2778 mg/kg; vit. B6: 1200 mg/kg; vit. B12: 6 mg/kg; vit. C: 20,000 mg/kg; Niacin: 8000 mg/kg; Folic acid: 250 mg/kg; Biotine: 30 mg/kg; choline chloride: 100,000 mg/kg; Mn: 26,000 mg/kg; Fe: 10,000 mg/kg; Cu: 1,000 mg/kg; Zn: 15,000 mg/kg; I: 200 mg/kg; Se: 40 mg/kg. Excipient: calcium carbonate: 42.00%; spent grapes: 25.06%.

**Table 2:** The analytical composition of the five experimental diets supplemented with 0, 0.5 or 1% *Aurantiochytrium limacinum* for their whole life (WL; day 0–42) and for the fattening period only (FP; day 21–42).

Nutrient Value	Day 0–21 ^1^			Day 21–42		
	0%WL 0.5%FP 1.0%FP	0.5%WL	1.0%WL	0%WL	0.5%FP 0.5%WL	1.0%FP 1.0%WL
Dry matter (%)	89.4	89.0	89.2	88.9	88.8	88.9
Crude protein (%)	23.7	23.6	23.6	19.4	19.5	19.4
Crude fibre (%)	2.8	2.8	2.8	2.7	2.8	2.8
Crude fat (%)	6.5	6.5	6.9	6.8	6.5	6.9
Ash (%)	6.3	6.0	6.0	5.2	5.0	5.4
Starch (%)	40.2	40.5	39.9	43.5	43.9	43.5
Sugar (%)	4.6	4.6	4.6	4.5	4.6	4.6
M.E. ^2^ (Kcal/kg)	3180.5	3167.5	3169.0	3154.0	3158.0	3160.0

^1^ The crumbled feed provided from day 0–10 and pelleted feed provided from day 10–21 were analysed separately and so the values presented here represent the mean of these two analyses ^2^ Metabolisable energy calculated according to Gazzetta Ufficiale CE n. L54, February 22, 2009.

**Table 3:** The fatty acid composition of the five experimental diets supplemented with 0, 0.5 or 1% *Aurantiochytrium limacinum* for their whole life (WL; day 0–42) and for the fattening period only (FP; day 21–42).

Fatty Acid (mg/g)	Day 0–21 ^1^			Day 21–42		
	0%WL 0.5%FP 1.0%FP	0.5%WL	1.0%WL	0%WL	0.5%FP 0.5%WL	1.0%FP 1.0%WL
C12:0 Lauric	0.00	0.01	0.02	0.00	0.01	0.02
C14:0 Myristic	0.04	0.24	0.48	0.03	0.21	0.41
C15:0 Pentadecanoic	0.01	0.07	0.13	0.01	0.06	0.11
C16:0 Palmitic	6.24	7.91	10.47	6.44	7.65	9.89
C16:1 Palmitoleic	0.05	0.05	0.05	0.04	0.04	0.05
C17:0 Heptadecanoic	0.04	0.06	0.07	0.04	0.05	0.07
C18:0 Stearic	2.11	1.96	2.02	2.09	1.86	1.89
C18:1n9 cis Oleic	13.96	12.60	12.86	14.53	12.93	12.94
C18:1 cis11	0.61	0.55	0.56	0.59	0.52	0.52
C19:0 Nonadecanoic	0.11	0.10	0.10	0.11	0.09	0.10
C18:2n6c Linoleic	29.38	25.62	26.53	29.25	26.38	26.34
C18:3n3 α-Linolenic	2.27	1.95	1.98	2.20	1.94	1.96
C20:0 Arachidic	0.21	0.20	0.22	0.22	0.20	0.21
C20:1 cis-11-Eicosenoic	0.11	0.10	0.11	0.11	0.10	0.09
C20:5n3 EPA ^2^	0.00	0.00	0.00	0.00	0.00	0.00
C22:0 Behenic	0.18	0.17	0.18	0.18	0.16	0.16
C24:0 Lignoceric	0.08	0.08	0.08	0.08	0.07	0.08
C22:6n3 DHA ^3^	0.00	0.73	1.53	0.00	0.69	1.43

^1^ The crumbled feed provided from day 0–10 and pelleted feed provided from day 10–21 were analysed separately; the values presented here represent the mean of these two analyses ^2^ Eicosapentaenoic acid ^3^ Docosahexaenoic acid.

**Table 4:** The effect of dietary supplementation with 0, 0.5 or 1% Aurantiochytrium limacinum for their whole life (WL; day 0–42) and for the fattening period only (FP; day 21–42).

Parameter	0 %WL D 0–42	0.5%WL D 0–42	1.0%WL D 0–42	0.5%FP D 21–42	1.0%FP D 21–42	Standard Error	*p* Value
Weight Day 0	37.3	37.1	36.9	37.0	37.2	0.27	0.920
Weight Day 21	883.7 ^ab^	918.6 ^a^	848.3 ^b^	888.3 ^ab^	883.7 ^ab^	13.91	0.026
Weight Day 42	3072.1 ^a^	2998.1 ^ab^	2809.2 ^b^	2861.0 ^ab^	2947.8 ^ab^	59.10	0.028
^1^ ADG D 0–21	40.3 ^ab^	42.0 ^a^	38.5 ^b^	40.5 ^ab^	40.3 ^ab^	0.65	0.016
ADG D 21–42	101.9 ^a^	98.4 ^ab^	91.2 ^b^	93.9 ^ab^	97.5 ^ab^	2.52	0.051
ADG D 0–42	70.9 ^a^	70.0 ^ab^	64.8 ^b^	67.2 ^ab^	68.8 ^ab^	1.43	0.036
^2^ ADFI D 0–21	53.5 ^ab^	58.9 ^a^	53.4 ^ab^	53.0 ^ab^	52.6 ^b^	1.48	0.031
ADFI D21-42	162.9 ^a^	157.5 ^ab^	148.2 ^b^	151.2 ^ab^	153.2 ^ab^	2.95	0.014
ADFI D0-42	107.9	107.8	100.6	102.0	103.0	1.88	0.026
^3^ FCR D0-21	1.3	1.4	1.4	1.3	1.3	0.03	0.144
FCR D21-42	1.6	1.6	1.6	1.6	1.6	0.03	0.723
FCR D0-42	1.5	1.5	1.6	1.5	1.5	0.02	0.441
^4^ EPEF D21	317.5	315.0	287.6	323.7	322.8	9.01	0.049
EPEF D42	436.7	435.2	395.0	416.7	431.9	17.67	0.428
Breast weight (g)	604.6	590.3	556.6	552.1	575.0	20.39	0.388
Thigh weight (g)	581.4^a^	552.1 ^ab^	525.7 ^b^	509.3 ^b^	542.4 ^ab^	13.46	0.009
Dressing (%)	86.0	86.2	86.2	86.3	86.3	0.44	0.990
Breast (%)	23.4	23.6	23.0	23.2	23.2	0.67	0.980
Thigh (%)	22.5	22.1	21.8	21.4	21.9	0.48	0.551

^1^ Average Daily Gain; ^2^ Average Daily Feed Intake; ^3^ Feed Conversion Ratio; ^4^ European Production Efficiency Factor calculated as ((Average grams gained/day X % survival rate)/Feed Conversion X 10); ^a,b^ Means within a row that do not share a superscript differ significantly.

**Table 5:** The effect of dietary supplementation with 0, 0.5 or 1% Aurantiochytrium limacinum for the whole life (WL; day 0–42) or for the fattening period only (FP; day 21–42) on broiler tissue and organ fatty acid concentrations.

Fatty Acid (mg/100g)	0%WL D 0–42	0.5%WL D 0–42	1.0%WL D 0–42	0.5%FP D21–42	1.0%FP D 21–42	Standard Error	*p* Value
Breast							
C15:0	1.5 ^b^	2.2 ^ab^	3.2 ^a^	2.2 ^ab^	2.7 ^ab^	0.36	0.034
C20:5n3 EPA ^1^	1.9 ^b^	2.5 ^b^	4.3 ^a^	2.7 ^b^	3.5 ^ab^	0.39	0.001
C22:4n6	18.6 ^a^	10.9 ^b^	8.9 ^b^	11.7 ^b^	8.5 ^b^	0.87	<0.001
C22:5n3 DPA ^2^	12.1	11.3	12.4	11.5	11.7	0.76	0.841
C22:6n3 DHA ^3^	6.9 ^c^	32.6 ^b^	55.2 ^a^	30.3 ^b^	50.1 ^a^	2.26	<0.001
Total Omega 3	66.8 ^b^	83 ^ab^	111.9 ^a^	82.8 ^ab^	101.1 ^ab^	8.82	0.010
Total Omega 6	818.7	689.8	711.5	710.3	650.9	102.6	0.829
Omega 6 / Omega 3	12.2 ^a^	8.2 ^b^	6.2 ^c^	8.3 ^b^	6.3 ^c^	0.4	<0.001
Thigh							
C12:0	0.7 ^b^	1.1 ^ab^	1.1 ^ab^	0.8 ^ab^	1.2 ^a^	0.11	0.011
C14:0	14.6 ^b^	25.3 ^ab^	27.8^a^	19.4 ^ab^	30.0 ^a^	2.66	0.002
C15:0	3.0 ^c^	6.4 ^ab^	6.9 ^ab^	4.8 ^bc^	7.4 ^a^	0.59	<0.001
C17:0	5.8 ^b^	8.2 ^a^	7.0 ^ab^	6.9 ^ab^	8.1 ^ab^	0.58	0.040
C20:4n6	115.5 ^a^	97.8 ^b^	89.5 ^b^	100.1 ^ab^	91.6 ^b^	3.98	0.001
C20:5n3 EPA	2.9 ^c^	5.0 ^b^	8.2 ^a^	4.5 ^bc^	7.6 ^a^	0.44	<0.001
C22:4n6	25.8 ^a^	14.9 ^b^	11.9^b^	15.1 ^b^	11.7 ^b^	0.87	<0.001
C22:5n3 DPA	20.1	17.2	17.1	16.3	16.8	0.96	0.067
C22:6n3 DHA	11.7 ^c^	53.6 ^b^	86.0 ^a^	46.6 ^b^	79.6 ^a^	2.05	<0.001
Total Omega 3	140.7 ^c^	200.1 ^ab^	207.8 ^ab^	165 ^bc^	223.3 ^a^	13.3	0.001
Total Omega 6	1788	2087	1609	1695	1953	162.3	0.251
Omega 6 / Omega 3	12.7 ^a^	10.4 ^b^	7.7 ^c^	10.3 ^b^	8.6 ^c^	0.252	<0.001
Liver							
C15:0	1.5 ^b^	2.0 ^ab^	2.8 ^a^	2.2 ^ab^	2.8 ^a^	0.22	0.001
C20:4n6	346.8 ^a^	306.8 ^ab^	270.5 ^bc^	324.8 ^a^	257.2 ^c^	12.07	<0.001
C20:5n3 EPA	7.1 ^b^	10.0 ^b^	15.7 ^a^	10.7 ^ab^	15.3 ^a^	1.25	<0.001
C22:4n6	37.4 ^a^	24.7 ^bc^	21.5 ^bc^	26.5 ^b^	18.2 ^c^	1.8	<0.001
C22:5n3 DPA	27.7	23.6	26.9	29.2	24.1	1.79	0.153
C22:6n3 DHA	45.3 ^d^	161.7 ^c^	233.4 ^a^	187.6 ^bc^	214.4 ^ab^	8.82	<0.001
Total Omega 3	104.4 ^d^	215.9 ^c^	300.2 ^a^	251.4 ^bc^	281.5 ^ab^	11.16	<0.001
Total Omega 6	1143.9	1057.1	1074.1	1127.2	1085.2	59.31	0.820
Omega 6 / Omega 3	11.1 ^a^	5.0 ^b^	3.6 ^c^	4.5 ^bc^	3.9 ^bc^	278	<0.001
Kidney							
C20:2n6	35.0 ^a^	34.8 ^ab^	26.4 ^c^	32.3 ^abc^	27.9 ^bc^	1.72	0.002
C20:3n3	5.0 ^a^	5.2 ^a^	3.6 ^b^	4.8 ^ab^	4.2 ^ab^	0.33	0.010
C20:5n3 EPA	8.1 ^c^	25.5 ^b^	37.5 ^a^	26.4 ^b^	36.8 ^a^	2.3	<0.001
C22:4n6	24.2 ^a^	16 ^b^	14.2 ^b^	15.8 ^b^	11.3 ^b^	1.76	<0.001
C22:5n3 DPA	14.4	14.8	14.7	15.9	14.1	1.08	0.798
C22:6n3 DHA	21.2 ^c^	53.3 ^b^	70.8 ^a^	60.1 ^ab^	65.8 ^ab^	3.95	<0.001
Total Omega 3	122.3 ^b^	201.0 ^a^	192.6 ^ab^	180.7 ^ab^	193.8 ^a^	17.3	0.020
Total Omega 6	1798.1	2217	1600.5	1750.5	1710.6	256.43	0.506
Omega 6 / Omega 3	14.8 ^a^	10.8 ^b^	8.3 ^c^	9.3 ^bc^	8.7 ^bc^	0.591	<0.001
Skin with adhering fat							
C12:0	9.5 ^b^	11.6 ^ab^	15.0 ^a^	11.0 ^b^	13.1 ^ab^	0.95	0.004
C14:0	181.3 ^d^	248.5 ^bc^	332.7^a^	220.2^cd^	305.4 ^ab^	15.74	<0.001
C14:1	29.5	28.6	38.1	24.3	35.9	3.8	0.092
C15:0	34.6 ^d^	59.7 ^bc^	79.9 ^a^	51.8 ^c^	72.1 ^ab^	3.93	<0.001
C18:3n6	101.8	81.5	79.3	80.6	90.6	5.78	0.049
C20:4n6	251.2 ^a^	199.3 ^ab^	228.1 ^ab^	217.0 ^ab^	180.4 ^b^	16.35	0.047
C20:5n3 EPA	20.1 ^c^	30.7 ^bc^	71.6 ^a^	30 ^bc^	46.5 ^b^	6.05	<0.001
C22:4n6	57.5 ^a^	40.6 ^b^	41.6 ^ab^	40.9 ^b^	31.8 ^b^	4.02	0.002
C22:5n3 DPA	38.1	37.7	51.9	38.6	39.6	3.64	0.047
C22:6n3 DHA	18.5 ^c^	90.8 ^b^	210.1 ^a^	87.3 ^b^	147.3 ^b^	14.69	<0.001
Total Omega 3^4^	1372.9	1307.8	1448.6	1231.9	1345.2	85.44	0.489
Total Omega 6^5^	19460.0	18141.0	16840.9	16994.7	17248.3	1029.4	0.368
Omega 6 / Omega 3	14.2 ^a^	14.0 ^ab^	11.7 ^c^	13.8 ^ab^	12.9 ^bc^	0.317	<0.001

Means that do not share a superscript differ significantly. ^1^ Eicosapentaenoic acid; ^2^ Docosapentaenoic acid; ^3^ Docosahexaenoic acid; ^4^ Total Omega 3 = {C18:3n3+C20:3n3+C20:5n3+C22:5n3+C22:6n3}; ^5^ Total Omega 6 = {C18:2n6cis+C18:3n6+C20:2n6+C20:3n6+C20:4n6+C22:4n6}. ^a,b,c^ Means within a row that do not share a superscript differ significantly.

## Supplementary Materials

The following are available online at https://www.mdpi.com/2076-2615/9/6/327/s1. Table S1: The full fatty acid profile of breast, thigh, liver, kidney and skin (with adhering fat), taken from birds fed diets supplemented with A. limacinum at a rate of 0, 0.5 or 1% for their whole life (WL; day 0–42) or for the fattening period only (FP; day 21–42).
