# Supplementary material for: Whole-Life or Fattening Period Only Broiler Feeding Strategies Achieve Similar Levels of Omega-3 Fatty Acid Enrichment Using the DHA-Rich Protist, Aurantiochytrium limacinum
Source: Animals (Basel). 2019 Jun 6;9(6):327. doi: 10.3390/ani9060327 (PMC6617060; doi:10.3390/ani9060327)
Supplement: Supplementary file 1 [file animals-09-00327-s001.pdf]

animals

Supplementary Table 1.A

The effect of dietary supplementation with 0, 0.5 or 1% Aurantiochytrium limacinum for the whole life (WL; day 0–42) or for the fattening period only (FP; day 21–42) on broiler breast fatty acid concentrations.

Fatty Acid (mg/100g)	0 % D 0–42	0.5 % D 0–42	1.0 % D 0–42	0.5 % D21–42	1.0 % D 21–42	Standard Error	*p* value
C12:0	0.3	0.3	0.5	0.3	0.4	0.07	0.412
C14:0	7	8.1	12.3	8.2	10	1.56	0.155
C14:1	1.1	0.8	1.3	0.8	1	0.22	0.389
C15:0	1.5^{b}	2.2^{ab}	3.2^{a}	2.2^{ab}	2.7^{ab}	0.36	0.034
C16:0	424.4	371.8	437.1	364.6	394.4	55.32	0.856
C16:1	47.3	31	43.6	29.6	35.3	7.6	0.395
C17:0	2.9	3.1	3.5	3.3	3.1	0.45	0.921
C18:0	160.4	147.1	156.7	147.7	147.8	16.56	0.976
C18:1n9 cis	620.9	482.9	526.1	476.5	476.2	84.05	0.705
C18:1 cis11	36.4	29	30.2	28.6	29.2	3.29	0.435
C18:2n6c	709.5	600.4	625.5	615.3	565	98.01	0.878
C18:3n3	44.8	35.6	38.9	37.2	34.8	6.96	0.857
C18:3n6	3.9	2.9	3.3	3.3	3	0.56	0.710
C19:0	4.8	4.2	4	4.3	3.6	0.54	0.663
C20:0	1.7	1.5	1.6	1.5	1.4	0.22	0.951
C20:1	4.4	3.6	3.8	3.4	3.5	0.51	0.684
C20:2n6	10.4	6.8	9.7	9.6	9.3	0.72	0.893
C20:3n3	1.2	1.1	1.1	1.1	1.1	0.08	0.909
C20:3n6	8.9	7.7	8.9	8	8.1	0.67	0.626
C20:4n6	67.5	58	55.3	62.4	57	3.17	0.073
C20:5n3 EPA	1.9^{b}	2.5^{b}	4.3^{a}	2.7^{b}	3.5^{ab}	0.39	0.001
C22:4n6	18.6^{c}	10.9^{b}	8.9^{b}	11.7^{b}	8.5^{b}	0.87	<0.001
C22:5n3 DPA	12.1	11.3	12.4	11.5	11.7	0.76	0.841
C22:6n3 DHA	6.9^{c}	32.6^{b}	55.2^{a}	30.3^{b}	50.1^{a}	2.26	<0.001
Total Omega 3	66.8^{b}	83^{ab}	111.9^{a}	82.8^{ab}	101.1^{ab}	8.82	0.010
Total Omega 6	818.7	689.8	711.5	710.3	650.9	102.6	0.829
Omega 6 / Omega 3	12.2^{a}	8.2^{b}	6.2^{c}	8.3^{b}	6.3^{c}	0.4	<0.001

^{abc}Means that do not share a superscript differ significantly; Eicosapentaenoic acid (EPA); Docosapentaenoic acid (DPA); Docosahexaenoic acid (DHA); Total Omega 3 = {C18:3n3+C20:3n3+C20:5n3+C22:5n3+C22:6n3}; Total Omega 6 = {C18:2n6cis+C18:3n6+C20:2n6+C20:3n6+C20:4n6+C22:4n6}.

Supplementary Table 1.B

The effect of dietary supplementation with 0, 0.5 or 1% Aurantiochytrium limacinum for the whole life (WL; day 0–42) or for the fattening period only (FP; day 21–42) on broiler thigh fatty acid concentrations.

**Fatty Acid (mg/100g)**	**0 % D 0–42**	**0.5 % D 0–42**	**1.0 % D 0–42**	**0.5 % D21–42**	**1.0 % D 21–42**	**Standard Error**	***p* value**
C12:0	0.7[b]	1.1[ab]	1.1[ab]	0.8[ab]	1.2[a]	0.11	0.011
C14:0	14.6[b]	25.3[ab]	27.8[a]	19.4[ab]	30.0[a]	2.66	0.002
C14:1	2.4	3.0	3.3	2.2	3.4	0.41	0.163
C15:0	3.0[c]	6.4[ab]	6.9[ab]	4.8[bc]	7.4[a]	0.59	<0.001
C16:0	848.7	1030.5	934.8	832.1	1114.9	93.32	0.184
C16:1	107.5	118.7	116	89.2	133.1	16.25	0.428
C17:0	5.8[b]	8.2[a]	7.0[ab]	6.9[ab]	8.1[ab]	0.58	0.040
C18:0	296.5	342.4	297.6	292	343.5	21.57	0.237
C18:1n9 cis	1308.8	1494.7	1200.2	1187.6	1508.2	141.4	0.331
C18:1 cis11	67.7	71.1	59.5	59.8	72.6	5.79	0.356
C18:2n6c	1611.5	1936.3	1474	1547	1811.8	158.14	0.234
C18:3n3	104.5	122.7	95	96.2	117.7	10.97	0.291
C18:3n6	7.7	8.6	6.7	7.3	9.2	0.78	0.193
C19:0	8	8.9	7	7.1	8.6	0.63	0.151
C20:0	3.1	4	3	3.4	3.8	0.34	0.216
C20:1	8.3	9.5	7.5	7.4	9.5	0.79	0.188
C20:2n6	13.8	15.2	13	12.9	14.4	0.81	0.257
C20:3n3	1.5	1.6	1.4	1.4	1.6	0.09	0.392
C20:3n6	14	14.3	14	13	14.6	0.78	0.674
C20:4n6	115.5[a]	97.8[b]	89.5[b]	100.1[ab]	91.6[b]	3.98	0.001
C20:5n3 EPA	2.9[c]	5.0[b]	8.2[a]	4.5[bc]	7.6[a]	0.44	<0.001
C22:4n6	25.8[a]	14.9[b]	11.9[b]	15.1[b]	11.7[b]	0.87	<0.001
C22:5n3 DPA	20.1	17.2	17.1	16.3	16.8	0.96	0.067
C22:6n3 DHA	11.7[c]	53.6[b]	86.0[a]	46.6[b]	79.6[a]	2.05	<0.001
Total Omega 3	140.7[c]	200.1[ab]	207.8[ab]	165[bc]	223.3[a]	13.3	0.001
Total Omega 6	1788	2087	1609	1695	1953	162.3	0.251
Omega 6 / Omega 3	12.7[a]	10.4[b]	7.7[c]	10.3[b]	8.6[c]	0.252	<0.001

Means that do not share a superscript differ significantly; Eicosapentaenoic acid (EPA); Docosapentaenoic acid (DPA); Docosahexaenoic acid (DHA); Total Omega 3 = {C18:3n3+C20:3n3+C20:5n3+C22:5n3+C22:6n3}; Total Omega 6 = {C18:2n6cis+C18:3n6+C20:2n6+C20:3n6+C20:4n6+C22:4n6}.

Supplementary Table 1.C

The effect of dietary supplementation with 0, 0.5 or 1% Aurantiochytrium limacinum for the whole life (WL; day 0–42) or for the fattening period only (FP; day 21–42) on broiler liver fatty acid concentrations.

Fatty Acid (mg/100g)	0 % D 0–42	0.5 % D 0–42	1.0 % D 0–42	0.5 % D21–42	1.0 % D 21–42	Standard Error	*p* value
C12:0	0.3	0.3	0.4	0.3	0.4	0.05	0.319
C14:0	9.9	9.5	16.2	10	13.4	2.2	0.165
C14:1	0.7	0.5	1.1	0.6	1.0	0.24	0.384
C15:0	1.5[b]	2.0[ab]	2.8[a]	2.2[ab]	2.8[a]	0.22	0.001
C16:0	677.6	636.6	866.5	645.5	764	85.62	0.298
C16:1	27	17.4	32.7	22.1	32.1	6.15	0.362
C17:0	5.7	7.1	8.0	8.0	7.4	0.65	0.099
C18:0	614.3	598.5	695.3	612.7	600.8	48.92	0.612
C18:1n9 cis	526.1	445.1	641.1	489.3	576.7	101.88	0.693
C18:1 cis11	32.4	28	36	32.2	34.2	4.14	0.725
C18:2n6c	709.8	669.4	726.5	723.6	758.3	54.62	0.843
C18:3n3	22.8	18.7	22.5	22.1	26	3.24	0.640
C18:3n6	5.2	3.5	4.2	4.8	4.4	0.48	0.147
C19:0	5.4	5.1	5.3	5.4	5.6	0.34	0.897
C20:0	2.7	2.2	2.7	2.3	2.4	0.28	0.529
C20:1	5.8	5.8	6.9	5.4	6.4	0.98	0.844
C20:2n6	18.8	24	21.1	21.7	19.9	1.58	0.211
C20:3n3	1.4	1.9	1.7	1.7	1.7	0.16	0.353
C20:3n6	25.9	28.9	30.3	25.8	27.2	2.18	0.529
C20:4n6	346.8[a]	306.8[ab]	270.5[bc]	324.8[a]	257.2[c]	12.07	<0.001
C20:5n3 EPA	7.1[b]	10.0[b]	15.7[a]	10.7[ab]	15.3[a]	1.25	<0.001
C22:4n6	37.4[a]	24.7[bc]	21.5[bc]	26.5[b]	18.2[c]	1.8	<0.001
C22:5n3 DPA	27.7	23.6	26.9	29.2	24.1	1.79	0.153
C22:6n3 DHA	45.3[d]	161.7[c]	233.4[a]	187.6[bc]	214.4[ab]	8.82	<0.001
Total Omega 3	104.4[d]	215.9[c]	300.2[a]	251.4[bc]	281.5[ab]	11.16	<0.001
Total Omega 6	1143.9	1057.1	1074.1	1127.2	1085.2	59.31	0.820
Omega 6 / Omega 3	11.1[a]	5.0[b]	3.6[c]	4.5[bc]	3.9[bc]	278	<0.001

Means that do not share a superscript differ significantly; Eicosapentaenoic acid (EPA); Docosapentaenoic acid (DPA); Docosahexaenoic acid (DHA); Total Omega 3 = {C18:3n3+C20:3n3+C20:5n3+C22:5n3+C22:6n3}; Total Omega 6 = {C18:2n6cis+C18:3n6+C20:2n6+C20:3n6+C20:4n6+C22:4n6}.

Supplementary Table 1.D

The effect of dietary supplementation with 0, 0.5 or 1% Aurantiochytrium limacinum for the whole life (WL; day 0–42) or for the fattening period only (FP; day 21–42) on broiler kidney fatty acid concentrations.

**Fatty Acid (mg/100g)**	**0 % D 0–42**	**0.5 % D 0–42**	**1.0 % D 0–42**	**0.5 % D21–42**	**1.0 % D 21–42**	**Standard Error**	***p* value**
C10:0	0.9	0.9	0.7	0.8	0.9	0.099	0.565
C12:0	0.8	1	1	0.7	0.9	0.2	0.791
C14:0	16.1	23.6	23.9	17	23.4	4.39	0.541
C14:1	1.7	2	2.3	1.4	2.1	0.47	0.677
C15:0	4.6	6.9	7.1	5.5	7	0.9	0.277
C16:0	896.1	1066.8	946.3	836.8	994.1	153.91	0.855
C16:1	73.2	82.2	83.7	56.7	86.8	18.98	0.798
C17:0	9.7	12.4	11.1	11.6	11.5	0.78	0.210
C18:0	502.6	554.4	491.8	502.4	511.4	27.68	0.548
C18:1n9 cis	1115	1321.2	1051.3	957.1	1162	230.15	0.844
C18:1 cis11	64.2	66.4	56.9	44	61.5	8.86	0.420
C18:2n6c	1410.9	1880.1	1298.9	1420.9	1427.2	259.55	0.562
C18:3n3	73.6	102.2	66.1	73.5	72.9	17.77	0.647
C18:3n6	6	8.2	5.5	6.3	6.3	1.32	0.661
C19:0	7.7	9.2	6.9	8	8	0.84	0.456
C20:0	4.9	5.6	4.8	4.7	5.2	0.35	0.437
C20:1	9.2	10.3	8.2	7.8	8.9	1.06	0.527
C20:2n6	35.0^{a}	34.8^{ab}	26.4^{c}	32.3^{abc}	27.9^{bc}	1.72	0.002
C20:3n3	5.0^{a}	5.2^{a}	3.6^{b}	4.8^{ab}	4.2^{ab}	0.33	0.010
C20:3n6	43.6	42.4	42.2	44.6	34.6	2.87	0.137
C20:4n6	278.4	235.6	213.3	230.6	203.3	22.82	0.199
C20:5n3 EPA	8.1^{c}	25.5^{b}	37.5^{a}	26.4^{b}	36.8^{a}	2.3	<0.001
C22:4n6	24.2^{a}	16^{b}	14.2^{b}	15.8^{b}	11.3^{b}	1.76	<0.001
C22:5n3 DPA	14.4	14.8	14.7	15.9	14.1	1.08	0.798
C22:6n3 DHA	21.2	53.3	70.8	60.1	65.8	3.95	<0.001
Total Omega 3	122.3^{b}	201.0^{a}	192.6^{ab}	180.7^{ab}	193.8^{a}	17.3	0.020
Total Omega 6	1798.1	2217	1600.5	1750.5	1710.6	256.43	0.506
Omega 6 / Omega 3	14.8^{a}	10.8^{b}	8.3^{c}	9.3^{bc}	8.7^{bc}	0.591	<0.001

Means that do not share a superscript differ significantly; Eicosapentaenoic acid (EPA); Docosapentaenoic acid (DPA); Docosahexaenoic acid (DHA); Total Omega 3 = {C18:3n3+C20:3n3+C20:5n3+C22:5n3+C22:6n3}; Total Omega 6 = {C18:2n6cis+C18:3n6+C20:2n6+C20:3n6+C20:4n6+C22:4n6}.

Supplementary Table 1.

The effect of dietary supplementation with 0, 0.5 or 1% Aurantiochytrium limacinum for the whole life (WL; day 0–42) or for the fattening period only (FP; day 21–42) on broiler skin (with adhering fat) fatty acid concentrations.

**Fatty Acid (mg/100g)**	**0 % D 0–42**	**0.5 % D 0–42**	**1.0 % D 0–42**	**0.5 % D21–42**	**1.0 % D 21–42**	**Standard Error**	***p* value**
C10:0	8.4	6.7	8.6	9.3	7.9	0.697	0.130
C12:0	9.5^{b}	11.6^{ab}	15.0^{a}	11.0^{b}	13.1^{ab}	0.95	0.004
C14:0	181.3^{d}	248.5^{bc}	332.7^{a}	220.2^{cd}	305.4^{ab}	15.74	<0.001
C14:1	29.5	28.6	38.1	24.3	35.9	3.8	0.092
C15:0	34.6^{d}	59.7^{bc}	79.9^{a}	51.8^{c}	72.1^{ab}	3.93	<0.001
C16:0	9975.5	9473.2	10247.2	8500.5	10562.8	641.03	0.205
C16:1	1343.3	1151.3	1341	976.2	1398.8	145.33	0.238
C17:0	69.8	76.4	80.8	78.3	76.5	4.4	0.501
C18:0	2882.3	2768	2703.3	2611.7	2793.4	165.28	0.824
C18:1n9 cis	17184	15026.7	14615.6	13595.3	15794.8	1064.42	0.201
C18:1 cis11	696.2	584.7	559.9	531.9	627.4	44.34	0.106
C18:2n6c	18888.7	17664.7	16340.5	16512.4	16796.8	1009.26	0.380
C18:3n3	1287.1	1139.8	1106.9	1068	1103.5	74.27	0.283
C18:3n6	101.8	81.5	79.3	80.6	90.6	5.78	0.049
C19:0	69	60.1	59.7	60	58.4	3.53	0.237
C20:0	53.3	52.8	46.8	52.6	50.8	2.83	0.476
C20:1	106.1	98.9	88.4	85.5	100.6	6.37	0.146
C20:2n6	83.5	83.9	76.3	75.8	77	5.41	0.698
C20:3n3	9.1	8.8	8.2	8.1	8.3	0.59	0.713
C20:3n6	77.3	71	75.1	68	71.8	5.1	0.729
C20:4n6	251.2^{a}	199.3^{ab}	228.1^{ab}	217.0^{ab}	180.4^{b}	16.35	0.047
C20:5n3 EPA	20.1^{c}	30.7^{bc}	71.6^{a}	30^{bc}	46.5^{b}	6.05	<0.001
C22:4n6	57.5^{a}	40.6^{b}	41.6^{sb}	40.9^{b}	31.8^{b}	4.02	0.002
C22:5n3 DPA	38.1	37.7	51.9	38.6	39.6	3.64	0.047
C22:6n3 DHA	18.5^{c}	90.8^{b}	210.1^{a}	87.3^{b}	147.3^{b}	14.69	<0.001
Total Omega 3	1372.9	1307.8	1448.6	1231.9	1345.2	85.44	0.489
Total Omega 6	19460.0	18141.0	16840.9	16994.7	17248.3	1029.4	0.368
Omega 6 / Omega 3	14.2^{a}	14.0^{ab}	11.7^{c}	13.8^{ab}	12.9^{bc}	0.317	<0.001

Means that do not share a superscript differ significantly; Eicosapentaenoic acid (EPA); Docosapentaenoic acid (DPA); Docosahexaenoic acid (DHA); Total Omega 3 = {C18:3n3+C20:3n3+C20:5n3+C22:5n3+C22:6n3}; Total Omega 6 = {C18:2n6cis+C18:3n6+C20:2n6+C20:3n6+C20:4n6+C22:4n6}.
